# Cost-utility analysis of the screening program for early oral cancer detection in Thailand

**DOI:** 10.1371/journal.pone.0207442

**Published:** 2018-11-29

**Authors:** Chutima Kumdee, Wantanee Kulpeng, Yot Teerawattananon

**Affiliations:** 1 Health Intervention and Technology Assessment Program (HITAP), Ministry of Public Health, Taladkwan Subdistrict, Maung District, Nonthaburi, Thailand; 2 Saw Swee Hock School of Public Health, National University of Singapore, Singapore, Singapore; Massachusetts General Hospital, UNITED STATES

## Abstract

**Objective:**

To assess the cost-utility of an oral precancer screening program compared to a no-screening program in Thailand.

**Materials and methods:**

Markov models were performed to simulate costs and Quality Adjusted Life-Years (QALYs) of both the screening and no-screening programs in the Thai population aged over 40 years. There are four steps to the screening program in Thailand: 1) mouth self-examination (MSE); 2) visual examination by trained dental nurses (VETDN); 3) visual examination by trained dentists (VETD); and 4) visual examination by oral surgeons (VEOS). The societal perspective and lifetime horizon were applied. Variables used were derived from the pilot study of the oral precancer screening program in Roi Et province as well as through patient interviews and local and international literature reviews. Results were presented in terms of Incremental Cost-Effectiveness Ratios (ICER). Sensitivity analysis was performed to assess parameters uncertainty.

**Results:**

The screening program yielded higher costs (1,362 Baht) and QALYs (0.0044 years) than the no screening program, producing an ICER of 311,030 Baht per QALY gained. This indicates that the screening program is cost-ineffective in the Thai context, where the cost-effectiveness threshold is THB 160,000 per QALY gained. However, the programs will be cost-effective if the screening program are improved in one of three ways; 1) the sensitivity and specificity of MSE are more than 60%, 2) the sensitivity and specificity of VETDN are greater than 90%, or 3) the low accuracy steps like MSE or VETDN are removed from the screening program.

**Conclusion:**

The screening program is found to be cost-ineffective for oral precancer detection in Thailand. However, this study suggests 3 alternative policy options to ensure the cost-effectiveness of the program.

## Introduction

Oral cancer is one of 27 types of cancers in the world, with 263 new cases of oral cancer and 127 deaths per 100,000 in 2008 [1]. In Thailand, oral cancer incidence was 8.8 cases per 100,000 between 2007 and 2009. It mainly occurred in males and the average age of individuals diagnosed with oral cancer was 66.25 years [2]. In 2012, oral cancer prevalence was deemed the 6^th^ most common cancer in Thailand with an incidence rate of 188 per 100,000 population [3]. Based on this same rate, there would be approximately 5,700 new cases in 2015 [4].

The cause of oral cancer is not exactly clear. However, the major risk factors among the Thai population include tobacco use, alcohol consumption, and betel quid chewing. Smokers had a 1.8-fold (95% CI = 0.99 to 3.35) increased risk relative to those who had never smoked. Alcohol drinkers and betel quid chewers were 2.1 (95% CI = 1.19 to 3.68) and 4.1 (95% CI = 12.16 to 7.78) times more likely to develop oral cancer compared with those who were unexposed, respectively [5].

Overall, the median survival time of oral cancer in Thailand ranged between 0.07 and 2 years depending on the stage they were diagnosed at [6–9]. Treatment in the early stages, especially in oral precancerous or Potentially Malignant Disorder (PMD), has a great chance of being cured. On the other hand, untreated PMD can progress to oral cancer stage I, with the average time of 5.2 years after PMD diagnosis [10]. Visual inspection and palpation of oral mucosa under normal light by dentists or oral medicine specialists are widely used for PMD and oral cancer detection due to its ease of use and high accuracy [11]. Sensitivity and specificity of the detection are about 40–93% and 50–99%, respectively [12–16].

Early detection and diagnosis of PMD and oral cancer can improve a patient’s survival and quality of life. However, more than 80% of patients in Thailand were being diagnosed at the advanced stage [9]. Currently, there is no national oral cancer screening program in Thailand. Hence, the Bureau of Dental Health, Department of Health, Ministry of Public Health had designed a pilot sudy of oral precancer screening program properly to the Thai context; health-seeking behaviors, human resources, and budget. This pilot study was conducted in a rural area of Roi-Et province, Thailand aiming at assessing the accessibility and effectiveness of oral precancer screening program. Nevertheless, to consider the implementation of the oral cancer screening program, evidence of economic value is necessary. Therefore, this study was conducted at the request of policymakers along with the pilot study to assess the cost-utility of an oral cancer screening program compared to a no-screening program and to identify the design of the screening program that is most appropriate for Thailand regarding cost-effectiveness.

## Material and methods

### Study design

The study design is a Cost-Utility Analysis (CUA) using Markov models to simulate the oral precancer screening program, compared to the no-screening program among the Thai population aged over 40 years. Microsoft Excel 2016 was used to run the model with country-specific epidemiology, clinical parameters, and costs. Results were presented in terms of costs, Quality Adjusted Life-Years (QALYs), and Incremental Cost-Effectiveness Ratios (ICERs) by using the societal perspective—including both direct medical costs (i.e. screening, treatment, and rehabilitation costs) and direct non-medical costs (i.e. accommodation, transportation, food, and productivity loss). The model was run until all members of the cohort transited to the death state. Hence the future cost and outcome were discounted at 3% per annum [17].

### Model structure

A Markov model with a one-year cycle length was constructed to simulate a screening program and treatment of oral cancer for a lifetime horizon. Disease progression was identical for both the treatment and non-treatment groups, developing from no cancer to precancer and to oral cancer stages I, II, III, and IV, respectively. The modeled cohorts started at 40 years old from the no cancer state, and next cycle people will move to the pre-cancer stage with distribution based upon the oral cancer incidence in Thailand and adjusted by the probability of oral precancer from the literature review [2, 10]. After that, people in the cohort transited among cancer states based on transitional disease probability and were followed until they reached 100 years old or successfully moved to the death state.

**Fig 1:**
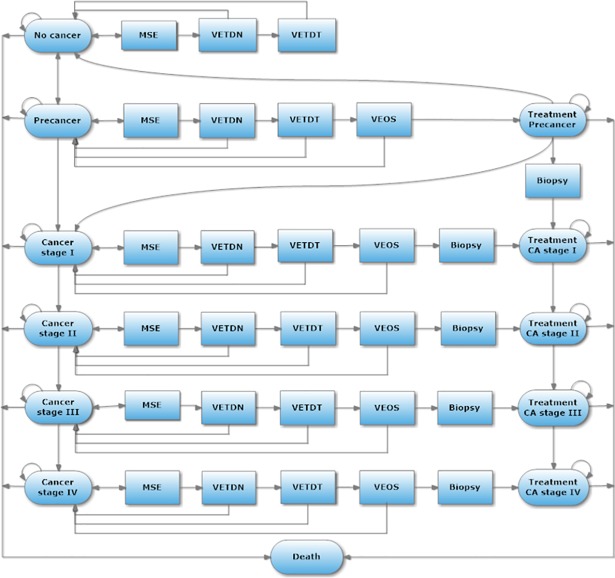
Model of the screening program for early oral cancer detection in Thailand.

The base-case program (scenario A) contains four main steps starting from Mouth Self-Examination (MSE), Visual Examination by Trained Dental Nurses (VETDN), Visual Examination by Trained Dentists (VETDT), and Visual Examination by Oral Surgeons (VEOS). In the MSE, the population aged over 40 years will receive documents educating on how to screen for oral lesions by themselves and questionnaires asking about their risk factors such as oral lesions, tobacco use, alcohol consumption, and betel quid chewing. Community health volunteers will distribute and collect the questionnaires at home. If people have checked that they found an oral lesion and had more than two risk factors in the step of MSE, this high-risk population will be referred to VETDN at primary care. People with a true positive and false positive results from VETDN will be screened through the next steps including VETDT at secondary, and VEOS at tertiary cares, respectively. However, each health state has a different final diagnostic confirmation–no cancer stage has identified by VETDT whereas pre-cancer stage has defined by VEOS. Oral surgeons will confirm people with true and false positive from VETDT and continue to the biopsy procedure for both the screening and the no-screening programs in order to confirm cancer diagnosis before getting into treatment. While others with true and false negative at every step of all screenings, they will remain the same health state during the cycle and they will be screened at MSE of again every year. (Fig 1).

To identify a cost-effective program for oral cancer detection in Thailand, four scenarios were introduced as follows:

Scenario A: Base case program

Scenario B: Removing MSE

Scenario C: Removing MSE and VETDN

Scenario D: Removing VETDN

All scenarios were compared to the no-screening program which has two general steps, i.e. the Visual Examination by General Dentists and VEOS, followed by the biopsy and treatment.

### Model input parameters

#### Epidemiological data

All parameters were listed in Table 1. The mortality rates of oral cancer in the treatment group were obtained by meta-analysis of survival studies in Thailand [7–9]. The non-treatment group had two times higher risk of death than those in the treatment group [9]. The death rate in oral precancerous patients both with and without treatment was assumed as being equivalent to age-specific mortality in the general population [18]. Disease progression in the non-treatment group was retrieved from international literature [10, 19]. It was assumed that disease progression in the treatment group would decrease by half compared to the non-treatment group in accordance with the risk of death which was compared between the non-treatment and treatment groups [9]. Patients who lost a follow-up of the oral precancer treatment can progress to cancer stage I with a probability of 0.01 –it was multiplied the probability of progression from precancer to cancer stage I (retrieved from the literature [19]) by the percentage of losing follow-up at oral precancer stage (taken from responses from the mail survey of Thai clinicians from 12 local hospitals). Oral precancerous lesions can be self-regressing to normal mucosa at 30% [20]. Thus, the probability of the treatment for pre-cancer stage to no cancer stage was calculated by multiplying the probability of self-regressing oral precancerous lesion by the probability of be cured from precancer treatment [21].

**Table 1:** Summary data and assumptions.

Parameter	Distribution	Mean	Standard Error (S.E.)	Reference
**Probability of disease progression**				
For no-treatment group				
Probability of progression from precancer to cancer stage I	Beta	0.04	0.01	[10]
Probability of progression from cancer stage I to II	Beta	0.53	0.27	[19]
Probability of progression from cancer stage II to III	Beta	0.59	0.25	
Probability of progression from cancer stage III to IV	Beta	0.67	0.25	
Probability of precancerous lesion regression	Beta	0.30	0.10	[20]
For treatment group				
Probabilities of disease progression of each cancer stage are less than the no-treatment group by a half. (using hazard ratio = 0.50)	-	0.50	-	[9]
Probability of precancerous lesion being cured after treatment	-	0.58	-	[20, 21]
Probability of loss to follow-up treatment and developing cancer stage I	-	0.01	-	[19] and mail survey
**Probability of death**				
For treatment group				
Probability of death from cancer stage I	Beta	0.20	0.01	Meta-analysis [7–9]
Probability of death from cancer stage II	Beta	0.34	0.06	
Probability of death cancer from stage III	Beta	0.42	0.08	
Probability of death cancer from stage IV	Beta	0.52	0.10	
For no-treatment group				
Probabilities of death of each cancer stage are more than the treatment group by a half. (using hazard ratio = 2)	-	2.00	-	[9]
**Compliance with screening**				
Screening program				
MSE at no cancer and precancer	-	0.97	-	Pilot study, by the Bureau of Dental Health
Visual screening by TDN at no cancer and precancer	-	0.80	-	
Visual screening by TDT at no cancer and precancer	-	0.76	-	
Visual screening by oral surgeons at precancer	-	0.91	-	
Visual screening at cancer stages I to VI	-	1.00	-	Assumption
Biopsy	-	0.62	-	S1 Table
No-screening program				
Self-referral rate to general dentist without precancer and cancer	-	0.04	-	[22]
Self-referral rate to general dentist at cancer stage I and II	-	0.43	-	
Self-referral rate to general dentist at cancer stages III and IV	-	0.82	-	
Biopsy	-	0.51	-	[23]
**Compliance with treatment**				
Precancer stage	-	0.94	-	Data collection
Cancer stage I	-	0.98	-	
Cancer stage II	-	0.98	-	
Cancer stage III	-	0.94	-	
Cancer stage IV	-	0.84	-	
**Performance of screening**				
Screening program				
Sensitivity of MSE	Beta	0.20	0.03	Pilot study, by the Bureau of Dental Health
Specificity of MSE	Beta	0.81	0.02	
Sensitivity of visual examination by TDN	Beta	0.44	0.06	
Specificity of visual examination by TDN	Beta	0.79	0.02	
Sensitivity of visual examination by TDT	Beta	0.87	0.04	[24–26]
Specificity of visual examination by TDT	Beta	0.85	0.05	[24–26]
Sensitivity of visual examination by oral surgeon	Beta	0.76	0.03	[27]
No-screening program				
Sensitivity of visual examination by general dentist	Beta	0.84	0.09	[28, 29]
Specificity of visual examination by general dentist	Beta	0.82	0.14	[28, 29]
Sensitivity of visual examination by oral surgeon	Beta	0.76	0.03	[27]
**Cost of screening**				
Direct medical costs				
Cost of screening program management	-	0.25	-	Pilot study, by the Bureau of Dental Health
Cost of MSE	-	5	-	[30] and data collection
Cost of visual screening by TDN	-	34	-	
Cost of visual screening by TDT	-	43	-	
Cost of visual screening by general dentist	-	95	-	[31]
Cost of visual screening by oral surgeon	-	90	-	[30] and data collection
Cost of biopsy	-	450	-	[32]
Direct non-medical costs				
Transportation cost of visual screening by TDN	Gamma	57	4	[33]
Transportation cost of visual screening by TDT	Gamma	76	4	
Transportation cost of visual screening by oral surgeon	Gamma	151	12	
Lost productivity work time to visual screening by TDN	Gamma	14	4	
Lost productivity work time to visual screening by TDT	Gamma	52	6	
Lost productivity work time to visual screening by oral surgeon	Gamma	85	14	
**Cost of treatment**				
Direct medical costs				
Precancer	Gamma	758	177	Data collection
Cancer stage I	Gamma	63,546	10,250	[34]
Cancer stage II	Gamma	68,753	15,870	
Cancer stage III	Gamma	78,749	8,205	
Cancer stage VI	Gamma	106,529	5,978	
Direct non-medical costs				
Precancer	Gamma	11,044	1,841	Data collection
Cancer stage I	Gamma	18,460	5,630	
Cancer stage II	Gamma	75,896	14,422	
Cancer stage III	Gamma	54,300	10,497	
Cancer stage VI	Gamma	54,265	9,511	
**Utility**				
Precancer	Beta	0.83	0.02	Data collection
Cancer stages I to II	Beta	0.61	0.05	
Cancer stages III to VI	Beta	0.34	0.09	

#### Compliance rates

Compliance rates of the screening in the no cancer and precancer stage were acquired from the pilot study. Due to unavailable data on compliance with screening at cancer stages I to IV, an estimate of 100% was given by experts because of the obvious signs of the oral cancer lesions and severe symptoms in stages I to IV such as painful tongue or loose teeth with no apparent reason. For the no-screening program, the compliance rates with screening were retrieved from a literature which divided the rates into no cancer, precancer, cancer stages I to II, and cancer stages III to VI [22]. Compliance with biopsy in the screening program was assumed to be 0.83 times higher than compliance rate in the no-screening program as with a previous oral cancer screening work [23]. For both the screening and no-screening programs, the compliance rates of treatment also were obtained from the mail survey of 12 local hospitals.

#### Sensitivity and specificity

Sensitivity and specificity of the screening program were gathered from the pilot study for the no cancer and the precancer stages whereas a systematic review and meta-analysis were performed by using the R software in order to obtain the sensitivity and specificity of the no-screening program [24–29]. The screening performances of cancer stages I to IV were assumed to be 100% in both the screening and the-no screening programs, owing to the experts’ claims that lesions are easily detected.

#### Costs

This study considered the societal perspective, which included both direct and non-direct medical costs. All cost parameters were calculated for 2016 in Thai baht (THB), the mean exchange rate between the US Dollar and the Thai baht in October 2016 was 1 USD equals THB 34.62. Costs of the screening program included the cost of management, e.g. dental nurse and dentist trainings, questionnaires for MSE, and incentives of health workers involved in the steps of MSE, VETND, and VETD, they were obtained from the pilot study. While, wages of health workers in the no-screening program were retrieved form literature review [30, 31]. All steps of screening were included transportation costs and lost productivity costs of patients [33]. For disease confirmation, the cost of biopsy was considered from the cost of healthcare services for reimbursement in Thai public hospitals [32]. Costs of diagnosis, treatment, and follow up of patients was collected from Roi Et Hospital where the pilot study was conducted. Costs of oral cancer treatment were obtained from a prior study in Thailand [34]. The direct non-medical costs were collected by interviewing patients of each health state at Roi Et Hospital, Chonburi Cancer Hospital, and the National Cancer Institute. In total, there were 9, 24 and 20 patients interviewed in precancer stage, cancer stage I-II, and cancer stage III-IV, respectively.

### Ethical clearance

This study was approved by the Research Ethics Committees of Roi Et Hospital, Chonburi Cancer Hospital and National Cancer Institute. Researchers informed objectives, benefits, process, and other risks according the study to participants. Written informed consent was obtained from all participations for being included in the study.

### Outcomes

Outcomes were measured in terms of QALYs calculated by multiplying the life expectancy by utility value. Utilities were acquired using the Time Trade-Off (TTO) method which is a direct utility measurement with acceptable validity and practicality [35]. The subjects at the hospitals as mentioned earlier were asked to choose between two alternative options; 1) living with current conditions for 10 years and 2) living in full health for shorter time (choices from 0–9 years), in order to find an indifferent point among the two options or the point that the responders cannot choose between living longer with imperfect health and living shorter with prefect health. Formulation of the utilities’ calculation is shown in S1 Appendix and the survey data can be available upon request.

### Model validation and data analysis

A face validation was conducted to check whether the model structure was a simplification of reality, and to ensure whether the parameters used in the model were appropriate in the Thai context. Moreover, we performed an internal validation to check whether the model produced results logically by extrapolating our predicted survival beyond observed data from a survival study in Thailand. To determine whether the screening program is cost-effective, ICERs were calculated using the formula. According to a manual of health technology assessment in Thailand, the intervention would be cost-effective if the ICER is less than or equal to a ceiling threshold of THB 160,000 per QALY gained [17]. To capture uncertainties in the result, a probabilistic sensitivity analysis was performed with 1,000 rounds of Monte-Carlo simulations and the result of this analysis was presented as a cost-effectiveness acceptability curve. Furthermore, one-and two-way sensitivity analyses were carried out to examine the uncertainly of results caused by selected variables.

## Results

**Fig 2:**
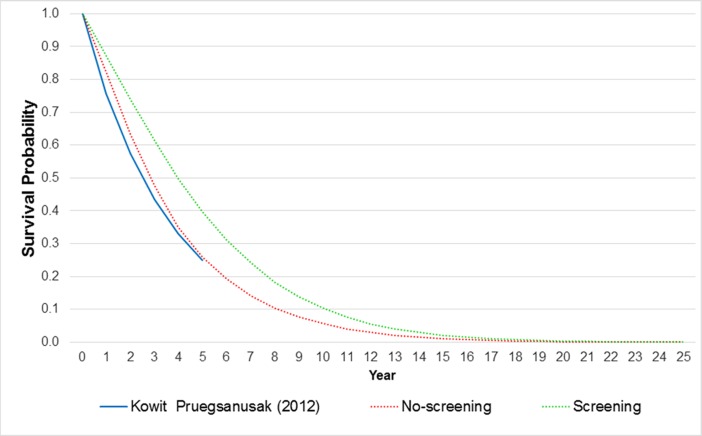
Model validation.

Fig 2 illustrates the comparison of predicted survival from our model and observed survival from a prior study in Thailand [9]. It was found that the predicted survival curve from the baseline model (no-screening program) was close to the curve from the observation study or real world data. Our baseline model yielded higher survival rates during the first four years; however, it produced a similar 5-year survival rate compared to the observed data.

Table 2 shows the incremental cost-effectiveness ratio (ICER) of oral cancer screening strategies. The screening program produced more costs (THB 1,362) and more effectiveness (0.0044 QALY) than the no-screening program. However, considering the country-specific threshold of THB 160,000 per QALY gained, the screening program was not cost-effective in the Thai context with an ICER of THB 311,030 per QALY gained.

**Table 2:** Lifetime costs and QALYs of screening versus no-screening.

Strategy	Cost (THB)	QALYs
Screening	2,765	21.6233
No-screening	1,403	21.6189
Increment between strategies	1,362	0.0044
**ICER**	**THB 311,030 per QALYs gained**	

**Fig 3:**
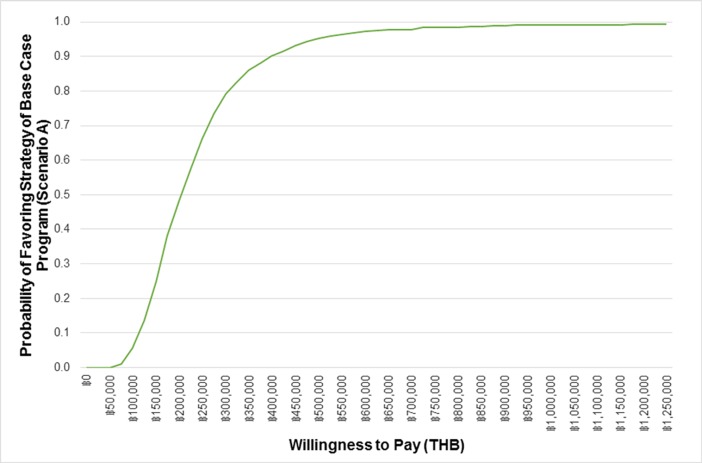
Cost-effectiveness acceptability curve.

Fig 3 presents the cost-effectiveness acceptability curve at any threshold. When considering the effects of parameter uncertainty, at the current threshold of THB 160,000 per QALY gained, the screening program had a 30% probability of being cost-effective compared to the no-screening program.

**Fig 4:**
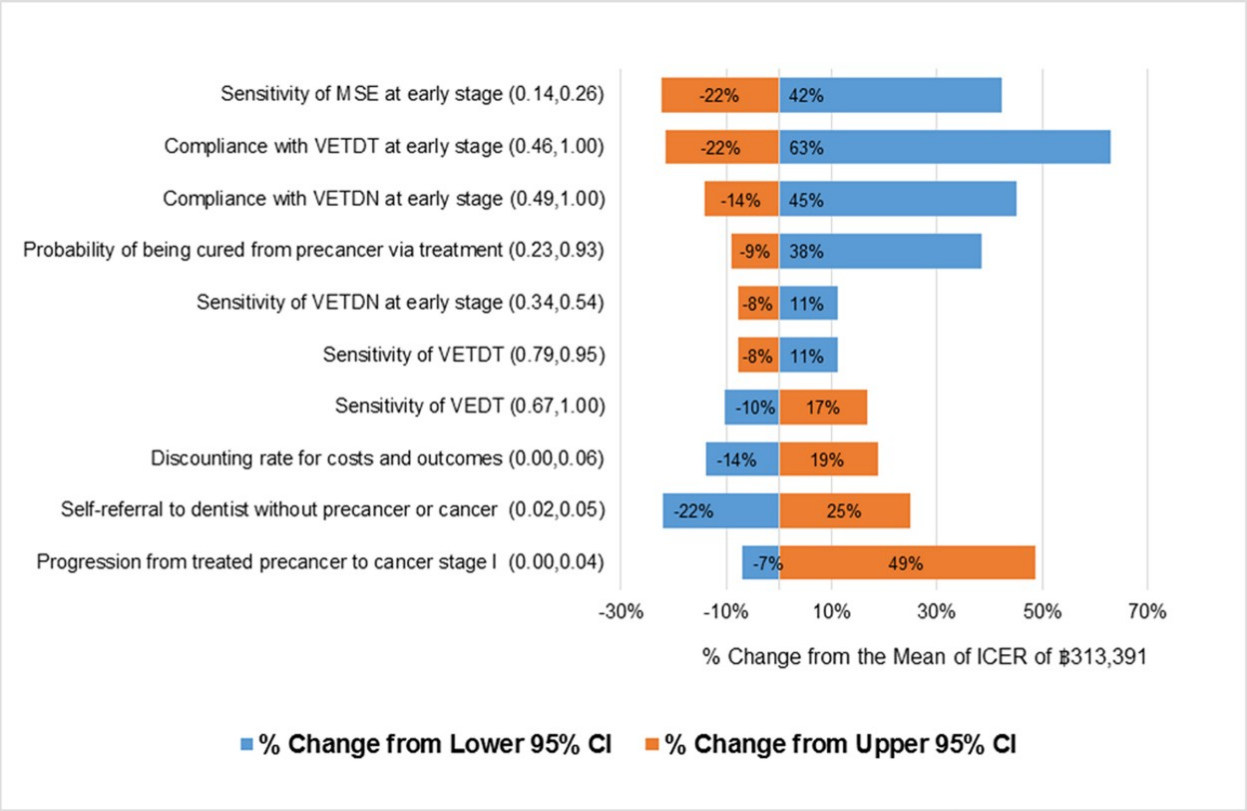
Tornado diagram of ICER sensitivity to plausible ranges of individual parameters.

Fig 4 presents the findings of the one-way sensitivity analysis, where all variables were varied between a low-to-high range based on the 95% confidence intervals (CI).Sensitivity of MSE for the early health state affected the deterministic mean of the ICER from changed to the upper 95% CI as well; after the adjustment also resulted in a 22%, decrease, the ICER was THB 250,556. Meanwhile, compliance with VETDT for the early health state had the highest dramatic effect on the cost-effectiveness result–when it was changed to the upper 95% CI, the deterministic mean of the ICER reduced by approximately 21%, coming out to THB 252,875. However, these drops in the deterministic means of ICER did not result in the program becoming more cost-effective. Moreover, when the compliance with VETDT and VETDN for the early health state were changed to the lower 95% CI; the screening program could not be accepted, it will be more cost-ineffective.

The two-way sensitivity analysis, where two variables were changed concurrently by 20%, demonstrates that the sensitivity and specificity of MSE and VETDN in early stage cancer affected the cost-effectiveness result. If the sensitivity and specificity of MSE were set at approximately 60%, the screening program becomes cost-effective with an ICER of 159,560 Baht per QALY gained. Likewise, 90% sensitivity and specificity of VETDN would be needed for the program to be cost-effective, with 160,322 Baht per QALY gained.

Table 3 illustrates the costs, QALYs, and ICERs of each scenario. Scenario A, which is the base case scenario of the screening program, produced an ICER of THB 320,618, resulting in the lowest QALYs gained. Scenario C, which removed MSE and VETDN from the base-case scenario, generated the highest cost and QALYs gained with an ICER of THB 100,016. Scenario D, which excluded VETDN, represented the most cost-effective scenario with an ICER of THB 82,292. However, the modifications of screening procedures in the three different scenarios produced ICERs under the cost-effectiveness threshold in Thailand.

**Table 3:** Summary of subgroup analysis.

Scenario	Steps of screening	Cost (THB)	QALYs	ICER (THB/QALYs)
**A (Base case scenario)**	1. MSE 2. Visual Examination by TDN 3. Visual Examination by TDT 4. Visual Examination by oral surgeon	2,616	21.6275	320,618
**B (Excluding MSE)**	1. Visual Examination by TDN 2. Visual Examination by TDT 3. Visual Examination by oral surgeon	4,498	21.6418	174,621
**C (Excluding MSE and visual examination by TDN)**	1. Visual Examination by TDT 2. Visual Examination by oral surgeon	5,199	21.6623	100,016
**D (Excluding visual examination by TDN)**	1. MSE 2. Visual Examination by TDT 3. Visual Examination by oral surgeon	2,938	21.6432	82,292

## Discussion

The screening program for oral precancer detection in Thailand seems to be cost-ineffective. Four steps are contributing to the screening program, and this leads to several major implications on program costs, effectiveness, and cost-effectiveness. First, the study shows that program compliance is the most important factor affecting the value for money of the screening program, which is related to a literature underlines the significance of screening acceptability is one of the key elements of quality assurance for every screening program [36]. The compliance rates of the screening program were obviously high due to strong response of the health workers in the setting to encourage people to screen at each step. Therefore, when the program is implemented nationwide, the compliance rates should be considered to remain stable at a high level by learning success from the pilot. Otherwise, the screening program might not be cost-effective as the data show in Fig 4. Second, the study indicates that two of the screening steps for early health state, i.e. MSE and VETDN, have relatively low screening accuracy which resulted in high cost and low QALYs gained. The screening program can be cost-effective if the sensitivity of MSE is improved from 20% to 60% and the specificity of MSE remains not less than 60%. In addition, the program will be cost-effective if the sensitivity and specificity of VETDN are improved from 44% and 79%, respectively, to 90% for both measures. Although, it may be possible that the development of better supporting tool and better trained community health volunteers who educate the target population for self-screening could improve the sensitivity of MSE to the preferable level, it is very unlikely that sensitivity and specificity of VETDN can enhance to 90% given the current performance of VETDT. As a result, this study estimated that if the low accuracy screening steps, i.e. MSE and VETDN, are removed from the program without compromising accessibility to screening by dentists among the target population, the screening program is also a preferable choice in terms of program effectiveness and cost-effectiveness.

The study indicates that the Scenario D, which includes MSE, VETDT, and VEOS, is considered the most feasible and practical choice under the Thai context because Thailand has a severe shortage of dentists (with 4,647 active dentists) at the public hospital to deliver screening services [37]. The inclusion of MSE aims to reduce the workload of dentists and make the program feasible on a national scale. Meanwhile, Scenario C, which excludes MSE and VETDN, offers the greatest QALYs gained because the target population would be seen directly by dentists, resulting in a much higher screening accuracy. However, it should be ensured that there is an enough number of dentists in public system to screen oral lesion while patients are at precancerous or early stages. For other countries without dentist accessibility issues should consider this option for a population-based oral cancer screening program. To sum up, these two screening options i.e. Scenario D and Scenario C, should be considered by countries aimed to introduce population-based oral cancer screening program with different contexts.

When comparing these results to other three cost-effectiveness studies previously published in academic journals, the community-based program of early oral cancer screening by trained health workers for population aged over 40 years in the United States yielded similar results, indicating poor value for money [23]. However, when changed to high-risk males aged over 40, the program is likely to be cost-saving. Moreover, the cost-effectiveness study of oral cancer screening in India was cost-effective [38]. This is because the screening program in India needs only one step, i.e. visual examination by healthcare workers, and targets high-risk population whose age is over 35 years and who use either alcohol or tobacco or both. The age standardized incidence rate (ASIR) of this Indian population is very high compared to the target population in Thailand, especially in males (12.6 VS 3.9 per 100,000) [39]. The study in the Netherlands confirmed that visual screening by specialists is cost-effective compared to the no-screening strategy; however, it is an opportunistic program rather than the population-based interventions investigated in the other three countries [40].

The key strength of this study is not only a health economic evaluation in Southeast Asia where oral cancer prevalence is moderate, but also the use of a new approach for threshold analysis to determine program characteristics that can make screening an optimal policy option in the Thai setting. These program characteristics can be used to further future research and development. On the other hand, this study has some limitations. Firstly, costs and performances of screening tools were collected from only one of total five sites due to budget constraints. Ideally, all variables used in the screening program should be completely collected prior to evaluation. Secondly, performances of the screening program received from the pilot study were overall proportion, unconsidered difficult between the screening of small and large lesions. Thirdly, certain variables were derived from international studies due to the lack of local epidemiological data. Nevertheless, oral cancer experts had examined all variables using the face validity method. In the future, we suggest conducting a clinical study to test the accuracy of screening tools and the feasibility of the modified program.

## Conclusion

The current oral cancer screening program for the Thai population aged above 40 years was not cost-effective. The screening can be cost-effective only if: 1) the sensitivity and specificity of MSE are more than 60%, 2) the sensitivity and specificity of VETDN are greater than 90%, or 3) the low accuracy steps like MSE or VETDN are removed from the screening program. Furthermore, these recommendations did not take into account the administration and management of the screening program such as workload, human resources, and implementation feasibility. Thus, policy makers should account for all various factors when implementing policy in a country-specific context.

## Supporting information

S1 Table Compliance rates of the oral precancer screening program in Thailand. (PDF)

S1 Appendix Time-trade off method. (DOCX)
