# Supplementary material for: Cost-utility analysis of the screening program for early oral cancer detection in Thailand
Source: PLoS One. 2018 Nov 29;13(11):e0207442. doi: 10.1371/journal.pone.0207442 (PMC6264816; doi:10.1371/journal.pone.0207442)
Supplement: S1 Table — (PDF) [file pone.0207442.s001.pdf]

S1 Table. Compliance rates of the oral precancer screening program in Thailand

Screenings	Total number of eligible participants	Number of compliances	Compliance rate
Mouth self-examination	9,750	9,482	97%
Visual examination by trained dental nurse	704	561	80%
Visual examination by trained dentist	561	429	76%
Visual examination by oral surgeon	22	20	91%
Biopsy	13	8	62%
