# Supplementary material for: Cost-utility analysis of the screening program for early oral cancer detection in Thailand
Source: PLoS One. 2018 Nov 29;13(11):e0207442. doi: 10.1371/journal.pone.0207442 (PMC6264816; doi:10.1371/journal.pone.0207442)
Supplement: S1 Appendix — (DOCX) [file pone.0207442.s002.docx]

**S1 Appendix. Time-Trade Off method**

Formulation for calculating utility


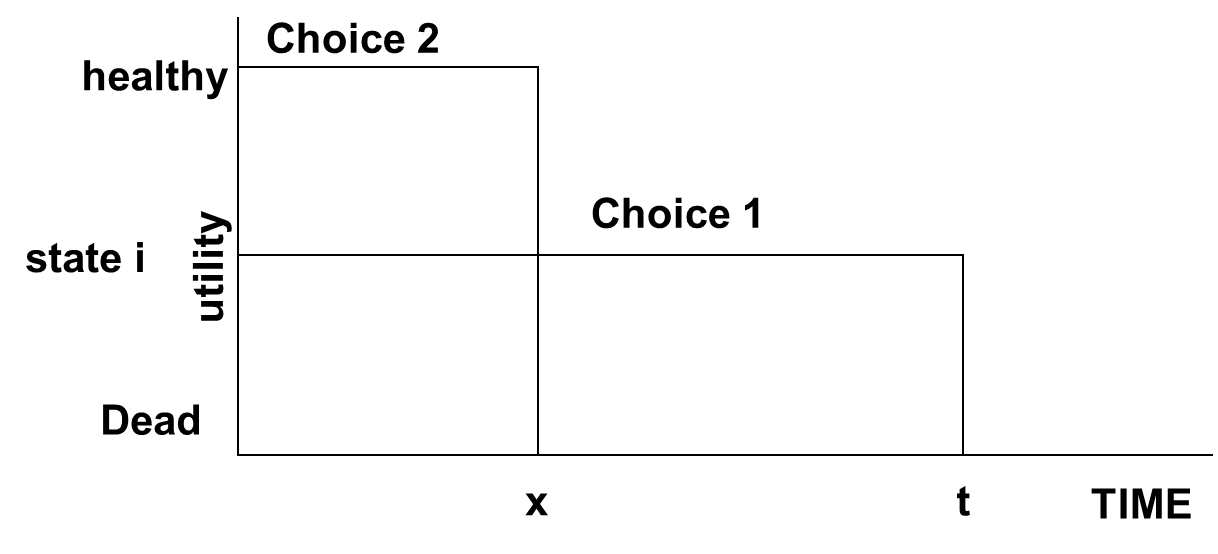


**U(i) = *x*/*t* (*x* ≤ *t*)**

U(i) stands for value of state i

i stands for imperfect health state

*x* stands for time in full health

t stands for time in state i
